# Multiscale analysis on otolith structural features reveals differences in ontogenesis and sex in *Merluccius merluccius* in the western Adriatic Sea

**DOI:** 10.1098/rsos.211943

**Published:** 2022-05-18

**Authors:** Quinzia Palazzo, Marco Stagioni, Steven Raaijmakers, Robert G. Belleman, Fiorella Prada, Jörg U. Hammel, Simona Fermani, Jaap Kaandorp, Stefano Goffredo, Giuseppe Falini

**Affiliations:** ^1^ Department of Chemistry ‘Giacomo Ciamician’, University of Bologna, Via Selmi 2, 40126 Bologna, Italy; ^2^ Laboratory of Fisheries and Marine Biology at Fano, Department of Biological, Geological and Environmental Sciences, University of Bologna, Viale Adriatico 1/N, 61032, Fano, Italy; ^3^ Computational Science Lab, University of Amsterdam, Science Park 904, 1098XH, Amsterdam, The Netherlands; ^4^ Marine Science Group, Department of Biological, Geological and Environmental Sciences, University of Bologna, Via Selmi 3, 40126 Bologna, Italy; ^5^ Institute of Materials Physics, Helmholtz-Zentrum Hereon, Max-Planck-Straße 1, Geesthacht, D-21502, Germany; ^6^ CIRI Health Sciences and Technologies (HST), University of Bologna, I-40064 Bologna, Italy; ^7^ Fano Marine Center, The Inter-Institute Center for Research on Marine Biodiversity, Resources and Biotechnologies, Viale Adriatico 1/N 61032 Fano, Italy

**Keywords:** *Merluccius merluccius*, Adriatic sea, ecomorphology, micro-CT scanning, functional morphology, sagitta

## Abstract

Otolith biomineralization results from biochemical processes regulated by the interaction of internal (physiological) and external (environmental) factors which lead to morphological and ultrastructural variability at intra- and interspecific levels. The aim of this study was to conduct a multi-scale analysis of the sagittal otoliths of the *Merlucius merlucius* (European hake) from the western Adriatic Sea in order to correlate otolith features with fish ontogeny and sex. We show that otoliths of sexually undifferentiated (non-sexed) individuals having a fish body total length (TL) less than 15 cm had faster growth in length, width, area, perimeter, volume and weight and a higher amount of organic matrix compared with otoliths of sexually differentiated individuals (females and males) having a fish size range of 15–50 cm. Most importantly, with increasing fish TL, female saccular otoliths contained a higher number of protuberances and rougher surface compared with male specimens, which showed more uniform mean curvature density. The differences between females and males discovered in this study could be associated with fish hearing adaptation to reproductive behavioural strategies during the spawning season. The outcomes of this research provide insights on how size and sex-related variations in otolith features may be affected by fish ecological and behavioural patterns.

## 1. Introduction

Otoliths, or ear stones, are three dense paired calcium carbonate (CaCO_3_) structures within a proteinaceous matrix, contained in three chambers associated with the inner ear of teleosts [1]. Otoliths act as mechanoreceptors involved in hearing through the detection of particle motion [2,3]. The size and shape of otoliths probably influence the frequencies that can be detected and the sensitivity (auditory threshold) to those frequencies [4]. Thus, the wide variability in ear morphologies and otoliths of fishes is probably linked to the diversity in hearing mechanisms and capabilities among different species [2]. Otoliths form during embryo development and continue to grow in incremental layers of CaCO_3_ in an organic matrix [5]. Consequently, otolith structure can also vary substantially during fish growth [6] in response to both physiological and ecological ontogenetic changes, and/or to differences in the acoustic environment related to diverse habitats occupied by juveniles and adults. Indeed, otoliths record the specifics of the physico-chemical environment experienced by a fish at any given point in its life and also provide information about its physiology related to ontogeny and feeding [7,8]. However, to date, only few investigations have focused on the relation between the morphological and ultrastructural differences of otoliths and the ecomorphological adaptations of the auditory system to habitat features such as water depth, feeding modalities, spatial niches and mobility [5,9–11].

The European hake (*Merluccius merluccius*) is a major component of the demersal fish assemblages and is distributed over a wide depth range (20–1000 m) throughout the Mediterranean Sea and the northeast Atlantic region [12]. The hake is an important predator of deeper-shelf, upper-slope Mediterranean communities. Previous studies, which were also conducted by experimental trawl surveys carried out in the Mediterranean [13], have observed a different bathymetric distribution during the ontogenesis of this species, while no differences were highlighted between females and males [14–16]. Juvenile hakes are mostly found around 170–220 m depth, intermediate hakes reach the highest abundance mainly on the continental shelf with a preference for shallower depths (70–100 m), especially when they reach 18–20 cm length [14,15,17]. Large hakes (greater than 36 cm) are found in a wide depth range but concentrate on the shelf break during the spawning period. Migration of juvenile hake from nursery areas on the shelf break and upper slope to the mid-shelf [17] is induced by a change in trophic requirements [18]. During its early life, the hake feeds on small crustaceans (Euphausiacea), where shrimp are among the most common preys in the muddy bottom communities of the Mediterranean Sea [15]. Subsequently, juvenile hakes migrate from the nursery areas to the parental stock, and when they reach a total length between 18 and 32 cm, they gradually change their diet towards pelagic and necto-benthic fish such as *Sardina pilchardus* and *Engraulis encrasicolus* [15,16]. These preys inhabit the coastal continental shelf and form schools usually deeper than 25 m [12]. Moreover, such trophic shifts coincide with an increase in the area of the inner ear of hake responsible for the detection and localization of objects, which takes place approximately at the critical size of 14–15 cm and could be important in detecting mobile prey such as fish [19]. Indeed, although hakes are demersal fishes, they feed typically upon fast-moving pelagic prey caught in mid-water or near the surface at night, undertaking daily vertical migrations [20,21]. Growth induces a continuous qualitative and quantitative change in diet that is reflected in the increasing mean weight of prey [15]. The shift toward large fish prey usually occurs slightly before maturity, the life-history stage with much higher energetic demands due to gonadal development [22]. Thus, increased energy demands related to sexual requirements, gonadal development and breeding activity appear to be the critical factors driving the changes in feeding strategy of *Merluccius merluccius*. Furthermore, in large hakes (greater than 36 cm), also cannibalism has been observed, probably in response to a great accessibility of conspecifics in the same area [23]. Nevertheless, most of the literature reports no difference in feeding habits between females and males [15,24].

The hake *M. merluccius* should be capable of vocalizing, as highlighted by the presence of paired drumming muscles for sound production located at the anterior wall of the swim bladder, similar to those found in known vocal fish species [25]. The same study also observed a sexual dimorphism in the drumming muscles during the spawning period of this species. In fact, only the drumming muscles from males are hypertrophied, while in females this effect is not observed, suggesting that adult males may increase the vocalizations in the context of spawning, like the males of other previously studied gadoids [25].

This multi-scale study investigated the sagittal otoliths characteristics of the *M. merluccius* from the central western Adriatic Sea. The first hypothesis is that different habitat distribution and feeding habits during the ontogenesis can leave a fingerprint in otolith characteristics that might provide clues related to hearing eco-functional adaptations to different environments and/or ecology during hake growth. To provide knowledge that could help in unravelling the challenging issue of ‘how sagitta morphology and structure varies regarding fish's ecological features and lifestyle (e.g. bathymetric distribution, habitat, feeding strategy and mobility pattern)?’ we performed an integrated comparison analysis of the morphometry, morphology and structure of otolith of sexually undifferentiated individuals (having gonads not macroscopically distinguishable and fish total length (TL) below the critical size of 15 cm) with data of sexually differentiated fishes (female or male with a size greater than 15 cm).

The second hypothesis concerns the sex-specific developmental pattern of the drumming muscles during the spawning season of hake previously seen in another study, which probably reflect different sound production associated with the calls in the reproductive behaviour of male with respect to female [25]. Consequently, it is reasonable to think that females exhibit auditory features capable of detecting the advertisement calls of males, since acoustic communication may play a crucial role in reproductive interactions [26]. Furthermore, the otolith features have an important role in fish hearing capabilities, and in particular, the morphology of otoliths is known to bring a functional significance. Although recent studies have focused on understanding the relationship between otolith features (e.g. biometry, morphology, density) and fish response to acoustic signals, [27–31], little is still known about the shape/structure-dependent otolith motion in response to harmonic waves. Therefore, it is interesting to compare otolith characteristics between conspecifics which share the same ecological context in order to exclude the otolith shape heterogeneity that can be determined by environmental, or/and ecological difference. In the context of the species investigated in this study, we performed an accurate description of the sagittae for sexually differentiated individuals (females versus males) to assess whether differences exist in otolith characteristics that could be related to hearing adaptation associated with acoustic communication in context of spawning.

The aims of this study were to: (i) correlate the morphometric (using two-dimensional image scanning programs), morphologic (by two-dimensional image scanning programs shape descriptors and micro-CT scans analysis), structural (through porosimetry technique) and compositional (by X-ray diffraction, thermogravimetric analysis and spectroscopy) otolith features with fish ontogeny and sex, (ii) verify whether there are any differences among undifferentiated, female and male otoliths which could be related to hearing adaptations to different habitats or behavioural contexts, and (iii) provide a new micro-CT scan-based approach developed using Python in combination with Visualization Toolkit libraries to investigate otolith shape curvature and perimetral irregularities (protuberances), displaying features not revealed with the canonical methods based on two-dimensional images.

## 2. Material and methods

Otoliths' biometry, two-dimensional contour, three-dimensional shapes, density and porosity, mineralogic composition, organic matrix content and incorporated elements were assessed. Furthermore, a new approach based on micro-CT scans was developed to investigate the overall curvature of otolith surface and to detect and count the protuberances of the contours, providing a methodological advancement toward the establishment of a reproducible, accurate and manual error-free measurement of three-dimensional otolith shapes. *Merluccius merluccius* was selected for the study for the following reasons: (i) it is a widely distributed, commercially important species in the Mediterranean Sea; (ii) otolith extraction more easily compared with other species, (iii) otolith data from other geographical areas in the Mediterranean Sea are available, (iv) it is a target species in which different methodologies have been applied, and (v) ecological and behavioural characteristics during ontogenesis and sexes make this species suitable to test adaptation to different habitat.

### 2.1. Sample collection

A total of 210 *M. merluccius* (61 non-sexed and 149 sexed) were collected from commercial catch, by benthic trawlers, longlines and gillnets, on May 2018 in the western Adriatic Sea, off the San Benedetto del Tronto coast (N 42°52′6.056″ E 14°33′43.29″, electronic supplementary material, figure S1). In the Mediterranean Sea, *M. merluccius* has three genetic clusters corresponding to the western, central and eastern Mediterranean populations [20,32,33]. Previous genetic studies based on molecular markers have not consistently defined a subdivision within western Adriatic hake stocks [34,35], therefore the samples used in this study were considered as belonging to the same fishing stocks.

For each specimen, fish total length (TL ± 1 cm) and weight (TW ± 1 g) were measured, and a macroscopic inspection of gonads was conducted to sex the fish. Sex categories were based on the sex maturity codes used by MEDITS-Handbook (2017) [36]. Undifferentiated or non-sexed fish showed inactive gonads. These fish are commonly referred to as juveniles [37] with less than 15 cm TL. Differentiated or sexed fish were 66 males (M) and 83 females (F) showing developed gonads and a body length greater than 15 cm TL.

Both sagittal otoliths were manually removed making a transverse cut with knife from the dorsal side of the fish head deep enough to reach the otic capsule. Then the head was flexed as if hinged near the snout, exposing the otic capsule and the otoliths which were then removed using forceps, cleaned from tissue with 3% H_2_O_2_ for 15 min and then washed with Milli-Q water, dried and stored inside Eppendorf microtubes. For the following analysis, the right otoliths were arbitrarily chosen since no scientific evidence suggests a side dimorphism in otoliths in this species [38].

### 2.2. Otolith biometry, morphology and structural parameters

Fish of a body length range of 6.9 to 45. 5 cm TL were included in the 210 otolith analyses based on digital images. These images were taken from a DCM 500 usb 2.0 5 MP linked to a Wild Heerbrugg M5A microscope. However, only 148 images of otoliths (40 non-sexed fish, 61 females and 47 males) were used to calculate structural parameters by buoyant weight. The relationships of otolith parameters with fish TL were determined for undifferentiated, females, males and for all the individuals combined (electronic supplementary material, tables S1–S2). Details on biometry, morphology and structural parameters are provided in the electronic supplementary material.

### 2.3. Otolith composition

For the mineral characterization of the samples analyses were conducted by X-ray powder diffraction (XRD) and Fourier transform infrared spectroscopy (FTIR). The otolith organic matrix and water content were assessed by thermogravimetric analysis (TGA) performed on powdered samples.

For the XRD analyses, the air-dried samples were ground in a mortar to obtain a fine and homogeneous powder (grains smaller than 100 µm) that was then loaded on a low background silica holder. XRD analyses were performed on 34 otoliths (eight undifferentiated, 13 females and 13 males) using a PANanalytical X'Pert Pro powder diffractometer equipped with X'Celerator detector (electronic supplementary material, figure S3).

FTIR analyses were conducted on samples previously used for the diffractometric analysis, by using a Nicolet FTIR 380 spectrometer working in the range of wavenumbers 4000–400 cm^−1^ at a resolution of 2 cm^−1^. This technique was used to confirm the X-ray powder diffraction data.

An estimation of the organic matter content for 35 powered samples (nine undifferentiated, 14 females and 12 males) was performed by TGA on a SDT Q600 simultaneous thermal analysis instrument (TA Instruments, electronic supplementary material, figure S4). Details on the XRD, FTIR and TGA procedures are provided in the electronic supplementary material.

### 2.4. Analysis of otolith microchemistry

Elemental analyses were conducted on powdered samples of six undifferentiated, nine females and nine males using induced coupling plasma-optical emission spectroscopy (ICP-OES). The analyses were performed on otoliths previously treated to remove surface contamination. Details are provided in the electronic supplementary material.

### 2.5. Morphological analysis on three-dimensional reconstruction of otoliths based on microcomputed tomography imaging

To investigate the three-dimensional shape of a subset of 24 otolith samples, high-resolution microcomputed tomography (micro-CT) scans were acquired with a GE Phoenix X-ray Nanotom S (electronic supplementary material, figure S5). The dataset consisted of six immature individuals and 18 adult samples, split in nine females and nine males having the same fish TL, in order to remove the impact of the different fish body size units and avoiding the standardization step. The isotropic voxel sizes in the scans varied from 2.024 to 8.333 μm depending on the actual size of the investigated otolith sample. Details on the procedure are extensively reported in the electronic supplementary material.

### 2.6. Statistical analysis

The relationships between otolith parameters (length, OL; width, OW; perimeter, OP; area, OA; circularity, OC; aspect ratio, OAR; roundness, OR; solidity, OS; volume, OV; weight, Oweight; micro-density, Omicro; bulk density, Obulk; porosity, Oporo; organic matrix, OM%) and TL were determined for undifferentiated, females, males and for all the individuals combined [39]. The best fits with the data to describe the relationships between otolith variables and fish somatic growth were first evaluated by curve estimation regression for three different curve models (linear, power and exponential, electronic supplementary material, table S3). When the best fitting was defined with nonlinear functions (power or exponential models) *y* = *ax^b^* or *y* = *a*e*^bx^*, ‘*y*’ is the otolith parameter, ‘*x*’ is fish length, ‘*a*’ is the factor and ‘*b*’ is the exponent. The parameters ‘*a*’ and ‘*b*’ were estimated through the linear regression analysis on log-transformed data: log (*y*) = log (*a*) + *b* log (*x*) (for power models) and log (*y*) = log (*a*) + *bx* (for exponential model). The relationships between otolith parameters and fish size were determined first for the entire group of individuals and then separately for undifferentiated, females and males, so that four growth curves were derived for each parameter (table 1, electronic supplementary material, table S4). The significance of the correlation was verified using Pearson's correlation coefficient. The statistical differences in regression slopes among groups were examined using a double approach to strengthen the analyses: comparing the confidence intervals of regression coefficients and checking the slopes of regression relationships through the analysis of covariance (ANCOVA). *Post hoc* tests after ANCOVA provided specific information on which regression lines were significantly different from each other in slope (table 1). Finally, principal component analysis (PCA) based on correlation matrix between groups was used to identify which otolithic biometric (length, width, perimeter), morphologic (circularity, aspect ratio, roundness, solidity, area) and structural (micro-density, porosity, bulk density, organic matrix content and initial temperature of degradation of CaCO_3_) parameters among the three otolith groups (undifferentiated, female and male) were more related to each other (electronic supplementary material, figure S11). Statistical analyses were performed using SPSS 20.0 and PAST 3 software.

**Table 1.** Regression parameters of the relationships between otolith biometric (OL = otolith length; OW = otolith width; OP = otolith perimeter; OA = otolith area) and morphologic parameters (OC = otolith circularity; OAR = otolith aspect ratio; OR = otolith roundness; OS = otolith solidity) with respect to fish size (TL = total length) of European hake for undifferentiated, females and males. P = power model, E = exponential model, L = linear model; *n* = sample size; *a* = constant; *b* = slope, CI (b) = 95% confidence interval; *R*^2^ = coefficient of determination; *p* = *p*-value. ANCOVA: Equality of slopes can be rejected when *p* < 0.05). *Post hoc* tests provide information on which regression lines were significantly different (≠) from each other in slope. Empty space indicates that the correlation was not significant.

relationship	fitting model	undifferentiated						females						males						ANCOVA	
		*n*	*a*	*b*	CI (b)	*R*^2^	*p*	*n*	*a*	*b*	CI (b)	*R*^2^	*p*	*n*	*a*	*b*	CI (b)	*R*^2^	*p*	homogeneity of slopes (b): *p*	*post hoc*
TL versus OL	P	61	0.021	1.174	1.105–1.235	0.959	<0.001	83	0.066	0.938	0.914–0.961	0.987	<0.001	66	0.083	0.900	0.860–0.940	0.970	<0.001	1.37 × 10^−08^	U ≠ (F = M)
TL versus OW	P	61	0.010	1.142	1.060–1.224	0.930	<0.001	83	0.038	0.872	0.840–0.904	0.973	<0.001	66	0.044	0.852	0.804–0.900	0.952	<0.001	3.37 × 10^−09^	U ≠ (F = M)
TL versus OP	P	61	0.029	1.307	1.240–1.375	0.961	<0.001	83	0.130	1.003	0.966–1.041	0.972	<0.001	66	0.280	0.861	0.811–0.910	0.950	<0.001	1.39 × 10^−15^	U ≠ F ≠ M
TL versus OA	P	61	0.000	2.354	2.230–2.482	0.959	<0.001	83	0.002	1.814	1.766–1.862	0.986	<0.001	66	0.003	1.742	1.666–1.820	0.970	<0.001	9.54 × 10^−15^	U ≠ (F = M)
OP versus OA	P	61	4.407	0.547	0.523–0.571	0.971	<0.001	83	4.430	0.550	0.529–0.569	0.987	<0.001	66	5.321	0.488	0.461–0.514	0.955	<0.001	7.70 × 10^−04^	(U = F) ≠ M
TL versus OC	P	61	1.839	0.261	(−0.374)–(−0.149)	0.268	<0.001	83	1.291	−0.193	(−0.264)–(−0.122)	0.264	<0.001	66	∕	∕	∕	0.003	>0.05	0.479	U = F
TL versus OAR	P	61	∕	∕	∕	0.001	>0.05	83	2.159	0.031	0.005–0.057	0.063	<0.05	66	∕	∕	∕	0.014	>0.05	∕	∕
TL versus OR	P	61	∕	∕	∕	0.001	>0.05	83	0.464	−0.031	(−0.057)–(−0.005)	0.064	<0.05	66	∕	∕	∕	0.014	>0.05	∕	∕
TL versus OS	P	61	∕	∕	∕	0.000	>0.05	83	0.914	0.010	0.007–0.14	0.318	<0.001	66	0.883	0.017	0.012–0.023	0.398	<0.001	0.029	F = M
TL versus OV	P	40	0.000	3.277	2.987–3.566	0.933	<0.001	61	0.000	2.368	2.283–2.454	0.981	<0.001	47	0.000	2.361	2.208–2.514	0.956	<0.001	5.59 × 10^−06^	U ≠ (F = M)
TL versus Oweight	P	40	0.000	3.225	2.976–3.473	0.948	<0.001	61	0.000	2.420	2.328–2.512	0.979	<0.001	47	0.000	2.437	2.277–2.596	0.977	<0.001	2.87 × 10^−06^	U ≠ (F = M)
TL versus Omicro	E	40	3.011	0.002	(−0.001)–(−0.000)	0.126	<0.05	61	2.668	0.0	0.000–0.000	0.696	<0.001	47	2.669	0.000	0.000–0.000	0.328	<0.001	1.00 × 10^−04^	U ≠ (F = M)
TL versus Obulk	E	40		∕	∕	0.026	>0.05	61	2.487	0.000	0.000–0.000	0.553	<0.001	47	2.411	0.000	0.000–0.000	0.307	<0.001	5.86 × 10^−05^	F ≠ M
TL versus Oporo	L	40		∕	∕	0.017	>0.05	61	∕	∕	∕	0.060	>0.05	47	9.700	−0.015	(−0.026)–(−0.005)	0.154	<0.01	∕	∕
TL versus OM%	P	9		∕	∕	0.029	>0.05	14	10.33	−0.355	(−0.565)–(−0.145)	0.531	<0.01	12	∕	∕	∕	0.268	>0.05	∕	∕

## 3. Results

### 3.1. Otolith biometry, morphology and structural parameters

Curve regression analyses (linear, power and exponential) were performed for testing the best fitting model for describing the general relationship for each dependent variable (parameters) with fish TL (electronic supplementary material, tables S3 and S4).

The results of the relationships between otolith length, width, area with fish TL among the three fish's groups showed differences (ANCOVA) between non-sexed and differentiated fishes, while no differences were highlighted between the females and males (table 1). The regression coefficients of undifferentiated fishes were significantly higher compared with males' and females' ones. There was significant difference in otolith perimeter–fish TL relationship among the three groups with a higher value of the regression coefficient in undifferentiated, followed by females and lastly the males' one.

Furthermore, the relation between otolith area and otolith perimeter showed a higher value of the regression coefficients in females' samples with respect to males' (table 1). For the circularity index, the correlation analysis with TL was significant only for undifferentiated and females and did not show differences in the regression coefficient between these two groups. The correlation analyses of the aspect ratio and roundness with fish TL were significant only in female. Concerning the solidity shape index, the correlation analysis was significant in females and males and the *post hoc* test did not show differences in the regression slopes between the two sex categories. The relationships between otolith volume with fish TL showed that the regression coefficients of undifferentiated fishes were significantly higher compared with males' and females' ones, while no differences were highlighted between the sexes (table 1).

Concerning the otolith structural parameters (micro-density, bulk density and porosity), otolith micro-density increased with increasing fish TL, from 2.64 g cm^−3^ at fish TL of 13.5 cm to 2.82 g cm^−3^ in individuals of 44.6 cm (table 2, electronic supplementary material, figures S4 and S6). Also, bulk density correlated positively with fish size, while porosity showed an opposite trend (electronic supplementary material, table S4 and figure S6). The bulk density correlated negatively with porosity while micro-density was positively correlated with bulk density (electronic supplementary material, figure S7).

**Table 2.** Mean structural parameters, the weight percentage values of the organic matrix (OM%) expressed in terms of weight loss (water + OM) and the initial temperature of the thermal decomposition of CaCO_3_, together with their standard deviations (s.d.) of undifferentiated, females, males and all the data pooled (total). The undifferentiated had greater weight loss than differentiated showing that the OM could have different roles depending on the ontogenesis. There was also a shift toward lower initial decomposition temperatures with increasing of fish TL, which could be related to a reduced energy barrier of the decomposition process and an increased amount of activated molecule. *n* = number of samples.

sex	*n*	fish TL (mm)	micro-density (mg mm^−3^)	bulk density (mg mm^−3^)	porosity (%)	*n*	fish TL (mm)	OM % (w/w)	initial temperature (C°)
		mean	mean ± s.d.	mean ± s.d.	mean ± s.d.		mean	mean ± s.d.	mean ± s.d.
undifferentiated	40	134	2.74 ± 0.09	2.55 ± 0.11	6.79 ± 2.92	9	142	1.99 ± 0.28	483 ± 37
females	61	280	2.77 ± 0.05	2.62 ± 0.08	5.31 ± 1.90	14	240	1.50 ± 0.67	451 ± 13
males	47	249	2.76 ± 0.04	2.60 ± 0.09	5.86 ± 2.38	12	234	1.48 ± 0.24	449 ± 13
total	148	231	2.76 ± 0.06	2.60 ± 0.09	5.88 ± 2.42	35	212	1.62 ± 0.32	457 ± 25

The content of organic matrix (OM wt%) decreased as fish TL increased (electronic supplementary material, figure S8; table 2, electronic supplementary material, table S4). Furthermore, a negative correlation between organic matrix content and both bulk density and micro-density was observed (electronic supplementary material, figure S9). The TGA profiles of most of the otolith samples contained two or three events with weight loss in the temperature range 130°C to 460°C (electronic supplementary material, figure S4). The initial temperature of decarbonation of CaCO_3_ is also reported (electronic supplementary material, figure S8; table 2) and there was a shift toward lower initial decarboxylation temperatures with increasing fish TL. No differences in otolith composition (100% aragonite) were found in undifferentiated, females and males (electronic supplementary material, figure S3). However, the measure of full width at half maximum (FWHM) values from the diffraction patterns showed a change in crystallite size with fish TL, with the presence of smaller crystallites in undifferentiated than in differentiated fishes (electronic supplementary material, figure S10).

Biplots of the principal component analysis (PCA) on the correlation matrix between the three groups representing the undifferentiated, female, and male otolith categories of the *M. merluccius* individuals are given in electronic supplementary material, figure S11. The first two axes (PC1 and PC2) of the PCA plots (electronic supplementary material, figure S11) showed a partial separation of otolith between the three groups representing the undifferentiated, female and male otolith categories of the *M. merluccius* individuals investigated. In particular, PC1 (electronic supplementary material, figure S11 a: approx. 84%, b: 79%, c: approx. 78%) separated the undifferentiated from differentiated through the otolith variables of length, width, area, perimeter, solidity, porosity, organic matrix content (OM%) and T° of CaCO_3_ decarbonation. Whereas PC2 slightly separated undifferentiated and males (which showed a wider overlapping area) otolith circularity, aspect ratio, bulk-density parameters from females (a: 16%, b: approx. 21%, c: approx. 22%).

### 3.2. Analysis of otolith microchemistry

In four out of six undifferentiated samples the concentration of trace elements resulted under the detection limits of the instrument not allowing the statistical analysis (electronic supplementary material, table S5). For nine females' and nine males' otoliths the concentration of 12 trace elements (Ba, Ca, Co, K, Li, Mg, Mn, Na, P, S, Sr and Zn) are reported in absolute concentrations (µg g^−1^, electronic supplementary material, table S6) and normalized to Ca (µmol mol^−1^, electronic supplementary material, table S7). Statistical analyses were conducted for each element but didn't reveal any differences in the quantitative analysis (ANOVA, *p* > 0.05, electronic supplementary material, table S6). Differently, the metal : Ca molar ratio values showed a significant difference in K/Ca and Na/Ca between sex (ANOVA, *p* < 0.05, electronic supplementary material, table S7). Moreover, a unique pool of individuals was then taken in account in this analysis (females + males; electronic supplementary material, figure S12). For all the elements, except K/Ca, Mn/Ca and Na/Ca, a negative correlation between the element and fish TL was observed, with a higher concentration of these elements in smaller adult sizes (electronic supplementary material, figure S12).

### 3.3. Morphological analysis on three-dimensional reconstruction of otoliths based on microcomputed tomography imaging

**Figure 1.**
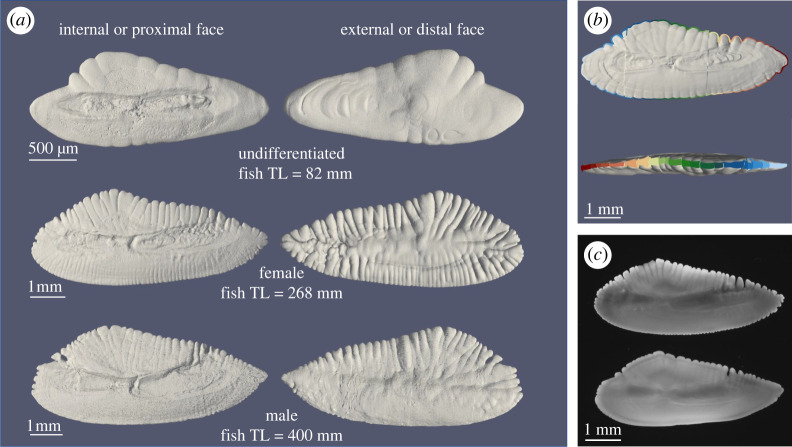
(*a*) Representative surface reconstruction of otolith's internal and external face by Marching Cubes algorithm visualized with ParaView. Fish TL means fish total length. (*b*) Representative images of detected protuberances (separated by colour) on proximal face (at the top) and sagittal plane (at the bottom) of a reconstructed otolith. (*c*) Otolith's sample of female (at the top) and male (at the bottom) of *M. merluccius.* The fish TL for both specimens is 300 mm. Otolith length is 14.6 and 14.4 mm in female and male, respectively. Note the difference in the shape and in the dentate protuberances along the perimeter (more pronounced in female). Otolith perimeter is 45.0 and 38.1 mm in female and male, respectively, while otolith area is almost the same between female (59 mm^2^) and male (60 mm^2^).

**Figure 2.**
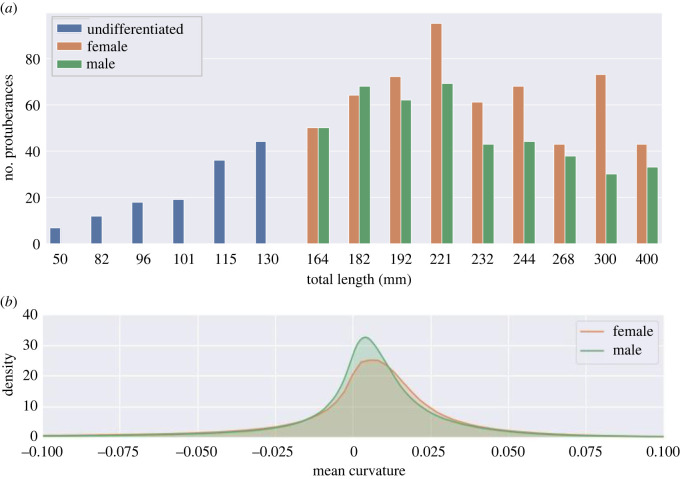
(*a*) Number of detected protuberances per fish total length for 24 samples. For females and males, a pairwise comparison of equal fish TL was performed. (*b*) Comparison of distribution plots by kernel density estimation (KDE) for *H* (mean curvature) on the surface for all female and male otoliths. The distribution for females contains considerably more positive *H* values in comparison with males (the female curve is shifted to the right). A comparison between distribution plots of *H* for males and females of equal fish length (see electronic supplementary material) shows the peaks of male otoliths to be consistently higher than its female counterparts. Consequently, the *H* values on male otoliths are more uniform, which indicates a smoother surface. This is also reflected by the difference in the number of detected protuberances.

The otolith three-dimensional reconstructions based on micro-CT imaging (figure 1, electronic supplementary material, figure S5) of non-sexed fishes showed fewer perimetric irregularities, no prominent branching-like protuberances, and a flat shape from the lateral view (figure 1, electronic supplementary material, figure S5). Instead, otoliths of sexed fishes showed a more elaborate structure, with a high number of irregularities on both the internal and external surfaces (figure 1, electronic supplementary material, figure S5). The curvature of the internal face tended to become more pronounced as the length of the fish increased, assuming an evident convex shape for the older samples (electronic supplementary material, figure S5). The number of otolith protuberances increased as fish grew (figure 2) and between females and males of equal fish TL the number of detected otolith protuberances was consistently higher for females. Peaks in the distribution *H* (mean curvature)—acquired via kernel density estimations (KDE)—for male otoliths were higher than those in females (figure 2). The peakedness (third moment of the density curve) decreased from top to bottom (less peaked shape) in female otoliths as fish grew (electronic supplementary material, figure S13).

## 4. Discussion

The study of eco-functional modifications in relation to changes in otolith features during fish growth is still at its infancy. In this study, we provided regression models describing the ontogenetic variation in otolith biometry, morphology, structural parameters (i.e. micro-density, bulk density, porosity, organic matrix content, crystallite size) and elemental composition of representative otolith samples from non-sexed and differentiated fishes of size range between 6.9 and 45.5 cm fish TL.

Under a same increase in fish TL, undifferentiated individuals (6.9 cm < TL < 15.0 cm) had a more pronounced increase in otolith length, width and perimeter compared with differentiated ones. We observed a higher concentration of organic matrix in otoliths of undifferentiated fishes compared with differentiated ones, which could explain the higher growth rates observed in the former compared with the latter. Indeed, the organic matrix contains various organic compounds (e.g. proteins, amino acids, collagens, proteoglycans) which are known to guide temporally and spatially the biomineralization process controlling and promoting the crystallites nucleation, orientation and growth [40–42]. Furthermore, the number of touching branch-like structures (referred to as protuberances) increased with fish TL. These differences suggest a heterogeneous distribution of the organic matrix, which can reflect a non-homogeneous deposition of CaCO_3_ along the surface of the otolith during fish growth [43]. Previous investigations performed on other species showed a decrease in otolith organic matrix content during fish ontogenesis and reported that the decrease in organic matrix content could be related to the change in trophic strategies [44,45]. Accordingly, the reduction in organic matrix content with fish growth, could be related to: (i) changes in feeding strategies and diet that occur during the life cycle of *M. merluccius* [8], (ii) reduction in feeding rates associated with energy demanding processes (e.g. sexual maturation and spawning) which could affect the biosynthesis of organic matrix macromolecules and their entrapment within the growing biomineral [46]. Since the mineralogic investigations have revealed uniform compositions (CaCO_3_ in aragonite form) regardless of fish TL, the decrease in otolith organic matrix with fish size could also explain the increase in micro-density from undifferentiated to differentiated fishes as the organic matrix has a lower density compared with aragonite [47]. Bulk density also increased with fish size, probably as the combination of increased micro-density and decreased apparent porosity. The ontogenetic variations in otolith biometric, morphological, structural and compositional parameters were also confirmed by the PCA analysis on the correlation matrix among the three otolith categories. Variations in otolith shape and structure during the ontogenesis can be associated with differences in terms of sound detections (structure–function relationship). The micromechanics of the tensors-associated masses (excrescences, roughness, furrows) of otolith shape and the otolith density may influence the acoustic stimulation and, consequently, modify the hearing capabilities in relation to fish size [27,30,48]. Otolith crystalline features also changed with fish size. Earlier studies have shown that the time-dependent distribution of a protein involved in the formation of otoliths (Starmaker-like protein) can have a significant effect on the crystallite size of growing crystals [42]. Therefore, the increment in aragonite crystallite size during otolith growth observed in *M. merluccius* could depend on variations in organic matrix composition [42]. Concerning otolith microchemistry, most of the investigated trace elements (element : Ca molar ratio) showed a negative correlation with fish size (Ba, Co, Li, Mg, P, S, Sr, Zn) which could depend on: (i) different water chemistry associated with water depth (Sr and Ba) [49], probably related to fish migration, and consequently (ii) shift in dietary sources (S) [50], and (iii) ontogenetic changes of the organic matrix content (P and Zn) [51]. Although most of the ontogenetic changes in otolith morphology and structure highlighted in *M. merluccius* could be the result of ecological adaptations to different habitats and/or trophic strategies, further acoustic and ecological studies must be carried out to assess the relations of structure–functionality associated with our observations.

Micro-CT imaging analysis resulted as a valuable approach to detect otolith protuberances and to quantify the amount of overall surface curvatures (ripples) of *M. merluccius* otoliths. The integration of the use of regression analysis of two-dimensional shape descriptors with a new method designated to analyse the otolith three-dimensional curvature from micro-CT images revealed for the first time a sexual dimorphism in the shape of sagittal otoliths in *M. merluccius*. Under a same increase in otolith area, females showed a higher increase in perimeter than males. The indices of circularity, roundness and aspect ratio with fish TL showed a higher amount of irregularities (dentate protuberances) in the contour and a more elliptical shape in otoliths from females compared with males. The morphological results obtained by the canonical two-dimensional image scanning programs were also corroborated by computational analyses based on micro-CT scans which highlighted the presence of a higher number of protuberances in the otolith of females in comparison with males of equal fish TL. In addition, the comparison of distribution plots by kernel density estimation (KDE) for *H* (mean curvature) on the otoliths surface of males and females of equal fish length showed that the peaks of male otoliths were consistently higher than its female counterparts. Consequently, the *H* values on otoliths were more uniform for males indicating smoother surfaces with respect to female, which instead were characterized by more wrinkled surfaces. Since no evidence of a spatial segregation between sexes has been reported so far [14–16], female and male fishes probably cohabit the same environment and are subject to the same exogenous factors. Therefore, the otolith shape dimorphism is probably less related to environmental factors and probably more influenced by genetically and physiologically controlled factors [6,52]. The differences highlighted between male and female otolith shapes may have a functional meaning linked to the sexual dimorphism of sound-generating muscles (drumming muscles) previously observed in this species [25]. Nonetheless, further studies aiming to establish the shape/structure–function relationships in otoliths are needed to confirm the hypothesis of an adaptive role in female's otolith related to the perception of male calls in the spawning context.

## 5. Conclusion

This study reports variations in otolith shape, morphology, structure and composition during hake (*M. merluccius*) ontogenesis. We revealed for the first time a sexual dimorphism in the otolith shape of hakes from the same geographical area by using a computational method developed to analyse otolith three-dimensional shape based on micro-CT scans.

The economic importance of hakes for European fishery makes this species subject of fish population studies in which establishing the sex of the specimens is a common practice. In such context, our study provides the basis for a new methodology for sex identification in hake specimens unrelated to gonadal inspection. This approach based on otolith sex dimorphism can be useful when fish gonadal tissues are unavailable due to damage or degradation (e.g. freezing), or to evaluate sex of preys from otoliths recovered from stomach of predators.

## Data Availability

The datasets generated during and/or analysed during the current study are available in the Figshare repository at the following URLs: https://figshare.com/s/5929597e5f699706a11b. https://figshare.com/s/0463cdd02b8faff29252. https://figshare.com/s/43a52c8bd9982477e8ca. Additional material is provided in the electronic supplementary material [53].
